# Investigating the content and correlates of undergraduate students' academic regrets

**DOI:** 10.3389/fpsyg.2025.1436323

**Published:** 2025-04-17

**Authors:** John Ranellucci

**Affiliations:** Faculty of Education, University of Ottawa, Ottawa, ON, Canada

**Keywords:** academic regrets, emotions, motivation, control-value theory (CVT), situated expectancy-value theory

## Abstract

**Introduction:**

The types of academic regrets that college students experience, characteristics of these regrets, and relations to motivational and emotional outcomes were investigated in two studies.

**Methods:**

Study 1 (*N* = 152) explored the relations between students' most severe academic regret and outcomes in general university courses, whereas Study 2 (*N* = 128) explored these relations in the context of a large introductory computer science course.

**Results:**

Across both studies, results suggested that students report various academic regrets. Generally, the types of regrets were unrelated to regret intensity, amounts of intrusive thoughts, or whether regrets were considered an omission or commission. Results further suggested that higher regret intensity was associated with motivational and emotional outcomes in the context of general university courses (Study 1), but not in the context of a specific undergraduate computer science course (Study 2).

**Discussion:**

Results are discussed within control-value theory and situated expectancy-value theory. Implications for practice are shared.

“You're not living if you're not regretting.”

Nicola Yoon

## Introduction

Experiencing some type of regret is an inevitable part of life. People may regret occupational choices such as becoming a professor, health related decisions such as drinking too much alcohol, or academic choices such as not choosing to do a bachelor's in computer science. Although regrets are an unpleasant emotional experience, they can help us reflect on past mistakes and shape future choices and behaviors. Reflecting on one's regrets can initiate corrective measures such as a change in career, healthier diet, or enrolment in a new academic program. As such, experiencing regrets can have an important function in the growth and development of healthy and happy individuals. Surprisingly, despite consistent evidence suggesting that the most common life regrets relate to education (Roese and Summerville, 2005) and a recent increase in research on emotions in education (Pekrun and Linnenbrink-Garcia, 2014), little work has investigated the relevance of regrets in educational contexts. Focusing on regrets can extend popular educational theories (e.g., affective memories within situated expectancy-value theory; Eccles and Wigfield, 2020) and can guide practice (e.g., past and anticipated regrets can help students make informed academic choices). Consequently, this paper identifies and ranks the categories of regrets that college students report, examines characteristics of these regrets, and explores relations with relevant motivational and emotional outcomes.

## Conceptualizing regrets

Regret is a negative emotion initiated by a counterfactual comparison with ones' current state and a desirable alternative state (Gilovich and Medvec, 1995; Roese and Olson, 1995; Zeelenberg, 1999). Experiencing regret requires an individual to reflect on a prior decision or event and to make a judgment about whether an alternative decision or event would have been better or led to more positive outcomes (Roese, 1997). Counterfactuals and regrets involve imagined alternatives to reality, the outcomes of which are not necessarily known (Gilovich and Medvec, 1995). Consequently, in contrast to other emotions, regrets can be considered “unusually cognitively-laden or cognitively-determined” (Gilovich and Medvec, 1995, p. 379).

Regrets can be broadly framed as either commissions (i.e., something done) or omissions (i.e., something not done). A regret of commission may involve regretting taking time off from school, whereas a regret of omission may involve not investing more effort in school. Although initially regrets of commission are often experienced as more intense, regrets of omission tend to be more troubling (Gilovich and Medvec, 1994, 1995), however some research has failed to replicate this finding (Richardson and Gilovich, 2023).

Research on regrets is often discussed under the umbrella of counterfactual thinking. Two popular theories of counterfactual thinking are norm theory and the functional theory of counterfactual thinking (Epstude and Roese, 2008). Within norm theory, regrets are depicted as a bias in judgment and decision making whereas the functional theory of counterfactual thinking conceptualizes regrets as a cognition about the past that is triggered following a failed goal, and which initiates a process to remedy the failure (Epstude and Roese, 2008). Empirical results offer a mix of support for both the cognitive biased and functional roles of regrets. For example, regrets predict lower levels of self efficacy and perceived competence (Carver, 2021), as well as adaptive decision making (O'Connor et al., 2015). Buchanan et al. (2016) offer one explanation for the mixed results by proposing that regrets consist of two factors, an affective and cognitive component, with only the latter being associated with functional outcomes. In addition to exploring the pathways from regrets to behavior, researchers have also explored the types of regrets that are commonly experienced.

Results from a meta-analysis of 11 regret ranking studies found that the most frequently reported life regret related to education (Roese and Summerville, 2005). Although regrets associated with education are common, little is known about the specific types of academic regrets that students experience. Regret ranking studies tend to group life regrets into broad categories such as education, career, romance, or parenting (Roese and Summerville, 2005), however little research has disaggregated subtypes of regrets within these domains. A deeper understanding of the specific types of regrets that students report, as well as what these regrets predict can extend education theory and guide the development of practical implications to help students resolve past or avoid future regrets.

## Regrets in educational contexts

The little research investigating regrets in educational contexts to date has adopted various theoretical, conceptual, and methodological perspectives. Theoretically, regrets have been framed within social identity theory (Jones et al., 2012), motivational interference theory (Grund et al., 2014; Kuhnle et al., 2014), attribution theory (Stupnisky et al., 2011), control-value theory (Di Leo et al., 2019), and self-determination theory (Goto and Kusumi, 2015). With these disparate theoretical frameworks comes distinct conceptualizations of regrets, which undermines the conceptual clarity and coherence of research in this area. Within the educational literature, regrets have been conceptualized as a type of self-regulatory impairment (Grund et al., 2014), a personality construct (i.e., regret proneness; Kuhnle et al., 2014), a component of job satisfaction (Kunter et al., 2011; see Baumert et al., 2008), and an emotion (Jones et al., 2012; Stupnisky et al., 2011). The conceptualization of regrets even differs between studies where regrets are considered an emotion. For instance, Stupnisky et al. (2011) conceptualized regrets as a discrete emotion that is distinct from guilt and shame whereas Di Leo et al. (2019) defined shame as “a feeling of guilt, regret, or sadness” (p. 132). This type of conceptual variability makes it difficult to systematically increase our understanding of regrets in educational contexts. Consequently, identifying theoretical frameworks that may be useful for guiding educational researchers' investigation of regrets in academic contexts may help bring some clarity to the field.

## Connecting academic regrets to theory

Due to the complementary features and unique strengths, this research is guided by control-value theory (CVT) and situated expectancy-value theory (SEVT) (e.g., Lauermann et al., 2017; Rubach et al., 2023). Both theories explicitly incorporate affective elements (e.g., discrete emotions and affective memories) and include constructs relevant to academic regrets such as expectancy for success, values, and achievement goals (Eccles and Wigfield, 2020; Elliot, 2005; Pekrun, 2006; Wigfield and Eccles, 2000). However, the theories differ in how thoroughly some of these constructs are described (e.g., value) and while both theories propose reciprocal paths, the causal direction toward emotions differs.

CVT provides a framework for understanding antecedents and outcomes of emotions within achievement contexts (Pekrun, 2006, 2018, 2021). This theory proposes that environmental factors such as instructional quality predict students' perceptions of control and value, which in turn predict emotions. Subsequently, emotions predict learning and achievement outcomes. Furthermore, reciprocal paths are proposed which link antecedents, emotions, and outcomes in a cyclical pattern of causation (e.g., Pekrun et al., 2014, 2017). Regrets could be conceptualized within CVT as retrospective outcome-related emotions because they are experienced based on a *post-hoc* (i.e., retrospective) appraisal of a specific event or experience (i.e., an outcome) and could be considered a retrospective negative-activated or negative-deactivated outcome emotion.

**Figure 1:**
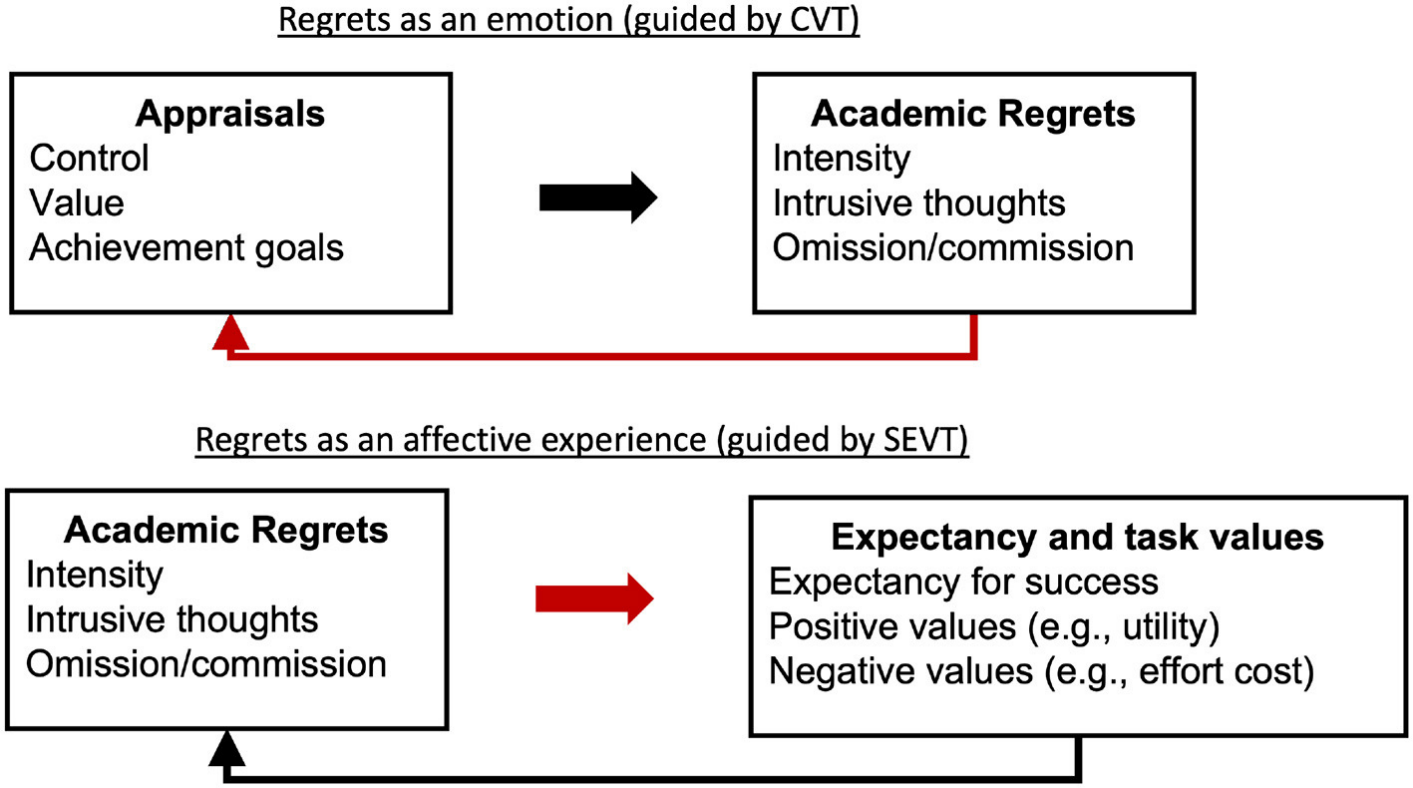
Conceptual model of academic regrets within control-value theory (CVT) and situated expectancy-value theory (SEVT). Red arrows indicate focal relations.

SEVT (Eccles and Wigfield, 2020) provides an additional explanation for the relevance of regrets in educational contexts, proposing that student's perceptions and interpretations of their social, cultural, and achievement related experiences shape their goals and self-schemata, and affective memories. In turn these goals, self-schemata, and affective memories predict students' expectations for success and task values, which subsequently predict achievement-related choices and engagement. Within SEVT, academic regrets can be considered a type of affective memory. Affective memories consist of emotional experiences associated with a past learning activity or event and are based on an individual's interpretation of previous achievement related experiences (Wigfield and Eccles, 2000). Pillemer (2001) notes that affective memories are based on significant life events that “continue to influence, inspire, and sustain actions and beliefs long after the original occurrence of the momentous events that they represent” (p. 124). This conceptualization provides a rationale for why a regretful event that may have occurred long ago can still have an impact on current thoughts, beliefs, and behaviors. However, in contrast to predictions based on CVT (i.e., appraisals of control, value, and achievement goals predict emotions), within SEVT, affective memories (e.g., academic regrets) are hypothesized to directly predict task values and indirectly predict expectancy for success through students' goals and general self-schemata (see Figure 1).

CVT and SEVT propose competence related beliefs (i.e., control or expectancy) and values as critical processes related to emotions such as regret. Expectancy for success consists of an individual's future focused competence related beliefs associated with a particular task (Wigfield and Eccles, 2000) and are quite similar to perceived control appraisals within CVT (Pekrun, 2006, see Marsh et al., 2019). Although there is some overlap between how values are conceptualized in both theories, SEVT provides a broader array of value components than CVT (Wigfield and Cambria, 2010), which is useful in the present study.

Within SEVT, task values can be separated into attainment value, intrinsic value, utility value, and costs (Eccles (Parsons) et al., 1983; Wigfield and Eccles, 2000). Attainment value involves the general importance of doing well. Intrinsic value refers to the enjoyment that an individual garners from engaging in a task, and utility value consists of the usefulness of the task in terms of one's future plans. Together, these represent positive values that should predict adaptive outcomes and behaviors such as persistence and choice. In contrast, cost represents more of a maladaptive type of value characterized by “negative appraisals of what is invested, required or given up to engage in a task” (Flake et al., 2015, p. 237). Although there are similarities between costs and regrets (e.g., both are negative appraisals with emotional and cognitive components), a critical distinction is that costs focus on perceptions associated with a particular task, whereas regrets focus on a downward counterfactual. For a cost-laden activity to relate to a regret, an individual must interpret an alternative outcome as more desirable. For example, reflecting on the psychological costs of a prior degree (e.g., stress and missed opportunities) may elicit pride for individuals who like their current job but may lead to regret for those who hate their current job. In the present study costs were separated into task effort, outside effort, loss of valued alternatives, and emotional costs according to the conceptualization forwarded by Flake et al. (2015). Task effort cost refers to the perceived time and effort that must be invested in the task. Outside effort cost refers to the time and effort associated with other competing responsibilities. Loss of valued alternatives costs entail the sacrifices or what must be given up to engage in the task, and emotional costs focus on the psychological toll that engaging in a task would involve. While SEVT provides a helpful elaboration of task values, CVT situates achievement goals as an additional construct that could be useful for understand students' academic regrets.

Achievement goals are the reasons for behavior in achievement situations (Ames, 1992) and are traditionally conceptualized according to a 2 × 2 taxonomy consisting of approach vs. avoidance and mastery vs. performance (Elliot and Murayama, 2008). Mastery-approach goals focus on developing competence, mastery-avoidance goals involve a focus on avoiding failure to reach ones' potential or to adequately develop, performance-approach goals are centered on demonstrating competence and outperforming peers, and performance-avoidance goals focus on a fear of doing poorly or being perceived as incompetent (see Hulleman et al., 2010). Within CVT, achievement goals are hypothesized to predict distinct patterns of emotional experiences (Pekrun et al., 2009), and therefore may relate to students' negative emotional experience of regret. Generally, mastery-approach goals negatively predict whereas performance-avoidance goals positively predict negative emotions such as anxiety, anger, boredom, and hopelessness (Pekrun et al., 2009; Ranellucci et al., 2015).

## Current study

Three research questions are explored in a domain general approach, where university students report motivation and emotions associated with their university courses in general (Study 1) and in a domain specific approach, where undergraduate students report motivation and emotions associated with an introductory computer science (CS) course (Study 2). Including a second study in a different context enables a conceptual replication, which focuses on the theoretical soundness and generalizability across general and specific academic contexts (Plucker and Makel, 2021) and considers the situated nature of motivation and emotion (Eccles and Wigfield, 2020; Pekrun and Marsh, 2022). Furthermore, a second study strengthens this largely exploratory project and since academic regrets are often associated with distal events that occurred months or even years prior to the outcomes being investigated (i.e., emotions, values, costs, and goals), examining general and specific academic outcomes is helpful for establishing boundary conditions for the results (Plucker and Makel, 2021). A CS course was selected because the subject does not overlap with the majors reported in Study 1 and therefore constitutes a distinct domain, and this course was a large lecture format which enabled data collection from a single course.

### RQ 1: What are the salient academic regrets reported by college students?

Research on regrets in academic contexts has focused on quantifying the amount or intensity of regret students report (e.g., Goto and Kusumi, 2015; Grund et al., 2014; Kuhnle et al., 2014; Stupnisky et al., 2011) however little research has examined the specific types of regrets that students may spontaneously report when asked to share their most severe academic regret. By assessing students' salient and most severe academic regrets, this study focuses on momentous experiences that form lasting and impactful affective memories. In turn, these affective memories are theorized to predict important motivational and emotional outcomes (Eccles and Wigfield, 2020; Wigfield and Eccles, 2000).

### RQ 2: How do salient academic regrets differ in terms of intensity, intrusive thoughts, and ratings of omission (i.e., something not done) or commission (i.e., something done)?

This research question builds on prior regret ranking studies by assessing intensity, intrusive thoughts, and ratings of regrets as omission or commissions (see Wrosch et al., 2007, 2005; Wrosch and Heckhausen, 2002). Based on the limited research that has investigated salient academic regrets to date it is difficult to predict characteristics of these regrets (e.g., the relative intensity). Consequently, no specific predictions are proposed for the intensity, amount of intrusive thoughts, and ratings of regrets as omission or commission.

### RQ 3: How do regret intensity, intrusive thoughts, and ratings of regrets as an omission or commission relate to achievement goals, expectancy, value, costs, and emotions?

Given the sparse research on academic regrets, we draw on CVT and SEVT to frame general predictions (Ames, 1992; Pekrun, 2006; Wigfield and Eccles, 2000). Within CVT, academic regrets would be conceptualized as negative activated or deactivated emotions, which emerged from prior appraisals (e.g., perceptions of control, value, and achievement goals), and through reciprocal paths predict subsequent appraisals. For instance, low perceived control may have contributed to a students' regrettable decision to not persist in a field, which in turn will have a negative impact on subsequent perceived control. Similarly, within SEVT, regrets could be considered a type of affective memory that should indirectly predict expectancy for success and directly predict task values and costs. Conceptualizing regrets as antecedents aligns with the temporal sequence of past regrets as affective memories theorized within SEVT that predict current processes, and the reciprocal relations among environment, appraisals, emotions, learning, and achievement proposed in CVT where regrets can be considered retrospective outcome emotions that predict subsequent appraisals and emotions (see red arrows in Figure 1). Based on these theoretical assertions, some tentative predictions can be proposed.

More intense regrets and regrets that involve more intrusive thoughts are hypothesized to predict more maladaptive outcomes, and less adaptive outcomes. Despite prior research highlighting the functional role of regrets, particularly when focusing on the cognitive component of regrets (Buchanan et al., 2016; Epstude and Roese, 2008), a maladaptive pattern of results is hypothesized based on the specific measures of regrets employed, including regret intensity, which is an affective component of regrets, and intrusive thoughts, which are unwanted, dysfunctional, and repetitive thoughts that are difficult to control (Epstude and Roese, 2008). Consequently, intensity and intrusive thoughts are expected to positively predict costs (i.e., task effort, outside effort, loss of valued alternatives, and emotional costs), avoidant goals (i.e., performance-avoidance and mastery-avoidance goals), and negative emotions (i.e., negative-activated and negative-deactivated). In contrast, intensity and intrusive thoughts are anticipated to negatively predict expectancy for success, task values (i.e., attainment, intrinsic, and utility), approach goals (i.e., performance-approach and mastery-approach), and positive emotions (i.e., positive-activated and positive-deactivated). Due to a lack of relevant empirical and theoretical research, no predictions are made for the relations between academic regrets as an omission or commission and achievement goals, expectancy, values, costs, and emotions.

## Study 1

### Participants and procedures

Participants were 152 college students recruited from a public urban university located in the northeast. Approximately 37% were undergraduate students, 61% were graduate students, and three students did not report their degree level. The mean age of participants was 26.85 (*SD* = 7.56). The sample consisted of 122 women, 25 men, one transgender student, and four students did not report their gender. Fifty-eight students identified as White and non-Hispanic, 31 identified as Hispanic or Latinx, 24 as Asian or Asian American, 23 as mixed-race, 11 as Black or African American, and five did not report their race or ethnicity. Approximately 69% of students were registered in the School of Education, 24% in the School of Arts and Sciences, one student was registered in the School of Nursing, two students reported an undeclared major, and five students did not report their majors.

Students who consented to participate in the study completed self-report questionnaires online at a single time point. Because there was concern that reflecting on regrets may influence the responses to subsequent questions, the only responses that participants provided after reporting their academic regrets were demographic questions. Participants completed the questionnaires for partial course credit as part of a research participation requirement for their courses or were compensated with a $10 online gift code. Results from qualitative content analyses (QCA) of open-ended regret responses indicated that nine students reported either “no-regret” or a “non-academic regret,” and five students reported regrets categorized as “competing commitments.” Because these two themes were either not relevant for the present study or represent very few participants, they were removed from subsequent quantitative analyses leading to a total sample size of 138.

### Measures

Participants were instructed to think about their current college experiences while answering the achievement goals, expectancy, value, costs, and emotions questionnaires.

#### Academic regrets

Academic regrets were assessed with an adapted version of a life regrets measure (see Wrosch et al., 2005, 2007; Wrosch and Heckhausen, 2002). Students were asked to write down their most severe academic regret in an open-ended question and to report whether the regret relates to a behavior that they have done (i.e., commission) or not done (i.e., omission).

Regret intensity was assessed by asking students to report the extent that they usually experienced negative affect (e.g., embarrassed) during the past few months associated with the regret noted (see Wrosch et al., 2007). An initial confirmatory factor analysis indicated that “sentimental” and “nostalgic” loaded poorly (<0.04) and were therefore removed from the scale. Responses were recorded on a Five-point Likert scale ranging from (1) not at all to (5) extremely and composite scores were created by averaging the six remaining items (α = 0.90).

Students were asked to rate how frequently they experienced types of intrusive thoughts associated with their regret on a scale ranging from (1) not at all to (5) often. A sample item is “I had trouble falling asleep because I couldn't stop thinking about the regret” (Wrosch et al., 2005). Composite scores were created by averaging the five items (α = 0.93).

#### Achievement goals

Mastery-approach, performance-approach, and performance-avoidance goals were measured using the Patterns of Adaptive Learning Survey (PALS; Midgley et al., 2000). Mastery-approach goals were assessed with five items, a sample item is “One of my goals in my classes is to learn as much as I can” (α = 0.85). Performance-approach goals were measured with five items, a sample item is “It's important to me that other students think I am good in my classes” (α = 0.89), and performance-avoidance were assessed with four items, a sample item is “One of my goals is to keep others from thinking I'm not smart in my classes” (α = 0.82). Mastery-avoidance goals were measured with seven items drawn from prior research based on face validity, a sample item is “It's important to me that I don't do worse than I know I'm capable of doing in my classes” (α = 0.81, see Baranik et al., 2007; Cury et al., 2006; Madjar et al., 2011).

#### Expectancy and task values

Expectancy for success was assessed using a five-item scale from the PALS (Midgley et al., 2000). A sample item is “I'm certain I can master the skills taught in my classes” (α = 0.87). Task values were measured with a scale adapted from Conley (2012) and Linnenbrink-Garcia et al. (2018). Attainment, intrinsic, and utility values were assessed with five items each. A sample attainment value item is “Being someone who is good at school is important to me” (α = 0.86). A sample intrinsic value item is “I am fascinated in my classes” (α = 0.95). A sample utility value item is “My classes will be useful for me later in life” (α = 0.87).

#### Costs

Four dimensions of cost were measured with Flake et al.'s (2015) cost scale. Task effort cost was assessed with five items. A sample item is “My classes are too much work” (α = 0.88). Outside effort cost was measured with four items, a sample item is “Because of other things that I do, I don't have time to put into my classes” (α = 0.90). Loss of valued alternatives was measured with four items, a sample item is “My classes require me to give up too many other activities I value” (α = 0.86). And emotional costs were measured with six items, a sample item is “My classes are emotionally draining” (α = 0.87).

#### Emotions

Emotions were measured using a scale adapted from Ben-Eliyahu and Linnenbrink-Garcia (2013). Positive-activated emotions were measured with five items (e.g., joyful; α = 0.87). Positive-deactivated emotions were assessed with three items (e.g., relaxed; α = 0.80). Negative-activated emotions were measured with five items (e.g., annoyed; α = 0.82). And negative-deactivated emotions were assessed with three items^1^ (e.g., bored; α = 0.88).

### Data analyses

Based on the design of this study, first participants' open-ended responses to the academic regrets question were analyzed, and then quantitative analyses were conducted based on the results from the coding as well as on the self-report scales.

#### Qualitative content analysis

QCA with an inductive coding procedure was used to identify and categorize the types of academic regrets that participants reported (e.g., Wrosch and Heckhausen, 2002). A coding manual with the code names, definitions, and examples was developed by two independent coders who individually created tentative codes and definitions based on 10% of the open-ended regrets reported and then met to refine the tentative manuals, a procedure that was iterated three times (see Supplementary Table 1S).

#### Quantitative analyses

Multivariate analyses of variance (MANOVAs), analyses of variance (ANOVAs), and chi-square analyses were used to determine if there were differences in intensity, intrusive thoughts, and ratings of regrets as an omission or commission based on the types of academic regrets identified in the qualitative content analysis (QCA). A series of multiple regressions were used to evaluate regret intensity, intrusive thoughts, and ratings of regrets as an omission or commission as predictors of achievement goals, expectancy, value, costs, and emotions.

## Results

### Preliminary analyses

Descriptive statistics, Cronbach alphas, and correlations are presented in Table 1. Skewness ranged from −1.268 to 0.778 and kurtosis ranged from −1.921 to 4.770, both within acceptable ranges for normality (e.g., Byrne, 2010; Hair et al., 2010). Confirmatory factor analyses (CFA) for all measures are reported in the Supplementary Table 6S.^2^ The overall pattern of correlations among achievement goals, expectancy, value, costs, and emotions is largely consistent with prior research.

**Table 1:** Correlations among all variables, study 1 and 2.

	**1**	**2**	**3**	**4**	**5**	**6**	**7**	**8**	**9**	**10**	**11**	**12**	**13**	**14**	**15**	**16**	**17**	**18**	**19**
*M*	4.18	3.84	4.17	4.21	2.99	2.90	3.02	2.95	2.77	3.91	4.21	3.18	3.61	2.98	3.52	4.11	–	1.85	2.58
*SD*	0.61	0.81	0.67	0.64	0.86	0.94	0.92	0.87	0.91	0.60	0.60	0.94	0.79	0.94	0.77	0.87	–	0.88	0.96
1 E	**0.87/0.88**	0.55^**^	0.58^**^	0.59^**^	−0.42^**^	−0.29^**^	−0.41^**^	−0.51^**^	0.43^**^	0.19^*^	−0.19^*^	−0.18^*^	0.32^**^	0.17	−0.15	−0.13	0.11	−0.31^**^	−0.23^**^
2 IV	0.72^**^	**0.95/0.90**	0.73^**^	0.65^**^	−0.43^**^	−0.33^**^	−0.46^**^	−0.37^**^	0.49^**^	0.29^**^	−0.09	−0.03	0.29^**^	0.05	−0.10	0.02	0.05	−0.13	−0.09
3 UV	0.68^**^	0.63^**^	**0.87/0.84**	0.67^**^	−0.30^**^	−0.19^*^	−0.31^**^	−0.22^**^	0.54^**^	0.39^**^	−0.12	−0.01	0.27^**^	0.03	−0.12	0.10	0.02	−0.18^*^	−0.17^*^
4 AV	0.58^**^	0.57^**^	0.80^**^	**0.86/0.88**	−0.28^**^	−0.27^**^	−0.33^**^	−0.23^**^	0.64^**^	0.45^**^	0.07	0.07	0.26^**^	0.01	−0.08	0.06	0.07	−0.06	0.12
5 TEC	−0.43^**^	−0.33^**^	−0.35^**^	−0.25^**^	**0.88/0.90**	0.56^**^	0.79^**^	0.75^**^	−0.16	−0.05	0.06	0.01	−0.28^**^	−0.23^**^	0.21^*^	0.19^*^	−0.06	0.24^**^	0.21^*^
6 OEC	−0.34^**^	−0.26^**^	−0.24^**^	−0.18	0.75^**^	**0.90/0.91**	0.62^**^	0.52^**^	−0.15	0.01	0.05	−0.01	−0.21^*^	−0.25^**^	0.25^**^	0.24^*^	−0.11	0.23^**^	0.26^**^
7 LoVA	−0.35^**^	−0.26	−0.28^**^	−0.20^*^	0.79^**^	0.86^**^	**0.86/0.88**	0.68^**^	−0.17^*^	−0.02	0.11	0.09	−0.28^**^	−0.23^**^	0.30^**^	0.22^*^	−0.08	0.22^**^	0.18^*^
8 EC	−0.53^**^	−0.48^**^	−0.41^**^	−0.28^**^	0.79^**^	0.66^**^	0.73^**^	**0.87/0.89**	−0.04	0.17^*^	0.26^**^	0.30^**^	−0.35^**^	−0.35^**^	0.36^**^	0.41^**^	−0.10	0.41^**^	0.31^*^
9 MAP	0.53^**^	0.54^**^	0.53^**^	0.41^**^	−0.32^**^	−0.35^**^	−0.40^**^	−0.37^**^	**0.85/0.79**	0.65^**^	0.11	0.11	0.18^*^	−0.04	0.05	0.18^*^	−0.02	−0.02	−0.12
10 MAV	0.29^**^	0.33^**^	0.29^**^	0.26^**^	−0.15	−0.13	−0.16	−0.20^*^	0.47^**^	**0.81/0.63**	0.35^**^	0.44^**^	0.04	−0.11	0.18^*^	0.22^*^	−0.04	0.18^*^	−0.01
11 PAP	0.07	0.11	0.14	0.17	−0.01	0.06	0.02	0.03	0.25^**^	0.28^**^	**0.89/0.86**	0.81^**^	−0.13	−0.02	0.17^*^	0.06	−0.15	0.29^**^	0.11
12 PAV	−0.05	−0.05	0.04	0.03	0.15	0.20^*^	0.14	0.16	0.11	0.25^**^	0.76^**^	**0.82/0.82**	−0.20^*^	−0.18^*^	0.18^*^	0.11	−0.08	0.31^**^	0.12
13 PA	0.46^**^	0.69^**^	0.44^**^	0.45^**^	−0.22^*^	−0.09	−0.11	−0.37^**^	0.29^**^	0.25^**^	0.04	−0.09	**0.87/0.92**	0.47^**^	−0.27^**^	−0.16	0.04	−0.22^*^	−0.17
14 PD	0.51^**^	0.44^**^	0.41^**^	0.30^**^	−0.41^**^	−0.32^**^	−0.34^**^	−0.57^**^	0.22^*^	0.20^*^	0.10	−0.07	0.55^**^	**0.80/0.82**	−0.26^**^	−0.39^**^	−0.11	−0.23^**^	−0.17^*^
15 NA	−0.27^**^	−0.30^**^	−0.19^*^	−0.16	0.50^**^	0.39^**^	0.45^**^	0.61^**^	−0.22^*^	−0.15	−0.03	0.09	−0.13	−0.40^**^	**0.82/0.78**	0.56^**^	−0.07	0.35^**^	0.18^*^
16 ND	−0.22^*^	−0.31^**^	−0.18	−0.14	0.48^**^	0.49^**^	0.55^**^	0.58^**^	−0.29^**^	−0.22^*^	−0.03	0.13	−0.21^*^	−0.27^**^	0.59^**^	**0.88/0.90**	−0.07	0.35^**^	0.18^*^
17 C/O	−0.04	−0.09	−0.10	−0.14	−0.11	−0.11	−0.10	−0.14	−0.10	0.07	−0.11	−0.11	−0.08	0.16	−0.11	−0.01	**–**	−0.15	−0.18^*^
18 RI	−0.07	0.10	−0.11	−0.12	0.04	0.12	0.09	0.11	−0.06	−0.05	0.06	0.15	0.12	−0.16	0.12	0.02	−0.21^*^	**0.90/0.90**	0.64^**^
19 IT	−0.07	0.13	−0.03	−0.09	0.11	0.06	0.02	0.12	0.01	−0.14	−0.10	0.03	0.07	−0.12	0.12	0.02	−0.12	0.62^**^	**0.88/0.91**
*M*	4.04	3.92	4.03	3.94	2.41	2.28	2.07	2.32	3.47	3.24	4.30	3.53	3.11	2.95	1.82	2.12	–	2.70	1.96
*SD*	0.74	0.74	0.67	0.83	0.73	0.81	0.67	0.81	0.84	0.97	0.66	0.84	0.94	1.04	0.72	0.99	–	1.03	0.89

Means, standard deviations, and correlations for study 1 above the diagonal, study 2 below the diagonal. Cronbach alphas bolded and on the diagonal, Study 1/Study 2. E, expectancy for success; IV, intrinsic value; UV, utility value; AV, attainment value; TEC, task effort cost; OEC, outside effort cost; LoVA, loss of valued alternative; EC, emotional cost; MAP, mastery approach goals; MAV, mastery avoidance goals; PAP, performance approach goals; PAV, performance avoidance goals; PA, positive-activated emotions; PD, positive-deactivated emotions; NA, negative-activated emotions; ND, negative-deactivated emotions; C/O, regret of commission (coded as 1) or omission (coded as 2); RI, regret intensity; IT, intrusive thoughts.

* *p* < 0.05;

** *p* < 0.01.

### RQ 1: Qualitative content analysis of open responses

Participants reported a range of academic regrets (see Supplementary Table 1S) which were categorized according to eight general themes based on the results of the QCA, (1) wrong class, program, or school (25.3%), (2) low effort persistence, or performance (22.7%), (3) poor timing (14.3%), (4) missed opportunity (11%), (5) not seeking help (9.7%), (6) a discrete behavior or decision (6.5%), (7) no regret/non-academic regret (5.8%), and (8) competing commitments (3.2%). The overall Cohen's Kappa = 0.936.

### RQ 2: Differences in intensity, intrusive thoughts, and ratings of omission/commission

Results from a MANOVA comparing ratings of regret intensity and intrusive thoughts by the regret theme coded in the QCA was non-significant, Wilks' Λ_(130, 2)_ = 0.946, *p* = 0.689, η^2^ = 0.028. This indicates that there were no mean differences in the intensity or the amount of intrusive thoughts among the types of regrets reported.

An ANOVA comparing ratings of intrusive thoughts by whether participants coded the regret as an omission or a commission was statistically significant, *F*_(1, 135)_ = 4.458, *p* = 0.037, η^2^ = 0.032, whereas the same ANOVA conducted on regret intensity yielded non-significant results, *F*_(1, 136)_ = 2.923, *p* = 0.090, η^2^ = 0.021. This suggests that participants who categorized their regrets as an omission (*M* = 1.718, *SD* = 0.835) reported slightly less intrusive thoughts associated with the regret than participants who categorized their regrets as a commission (*M* = 2.035, *SD* = 0.913). However, whether students consider their regret as an omission or commission did not relate to how intense students rated their regrets.

A 2 (ratings of omission or commission) × 6 (regret theme: missed opportunity; specific event or behavior; wrong class, program, or school; effort, persistence, or performance; timing; help seeking) chi-square test revealed a significantly non-random distribution of regret themes across ratings of omission [$\chi_{\left(5\right)}^{2}$ = 14.442, *p* < 0.013]. Investigation of the standardized residuals suggests that regrets associated with a missed opportunity were significantly more likely to be considered a regret of omission, no other differences were identified. Therefore, all regret themes included similar amounts of omission and commission regrets, except for regrets associated with a missed opportunity, which participants categorized as omissions more often than commissions.

### RQ 3: Predicting achievement goals, emotions, expectancy, value, and costs with regret intensity, intrusive thoughts, and ratings of omission/commission

Results from a series of multiple regressions, adjusted for multiple comparisons,^3^ can be found in Tables 2, 3 (see Supplementary Tables 2S, 3S for results when controlling for age, gender, and academic level). For expectancy, values, and costs, results indicated that ratings as a commission or omission, regret intensity, and intrusive thoughts explained a significant amount of expectancy [*R*^2^ = 0.101, *F*_(3, 132)_ = 4.938, *p* = 0.0028] and emotional cost [*R*^2^ = 0.168, *F*_(3, 133)_ = 8.980, *p* < 0.001], but did not relate to interest value [*R*^2^ = 0.018, *F*_(3, 132)_ = 0.813, *p* = 0.489), utility value [*R*^2^ = 0.037, *F*_(3, 132)_ = 1.707, *p* = 0.169], attainment value [*R*^2^ = 0.017, *F*_(3, 133)_ = 0.746, *p* = 0.526], task effort cost [*R*^2^ = 0.063, *F*_(3, 133)_ = 2.966, *p* = 0.034], outside effort cost [*R*^2^ = 0.078, *F*_(3, 133)_ = 3.748, *p* = 0.013], or loss of valued alternatives [*R*^2^ = 0.051, *F*_(3, 133)_ = 2.359, *p* = 0.074]. Only emotional intensity was a statistically significant predictor of emotional costs at the adjusted *p*-value cut-off (β = 0.351, *p* < 0.001) and although not statistically significant, emotional intensity negatively predicted expectancy for success (β = −0.265, *p* = 0.015).

**Table 2:** Study 1: predicting expectancy, values, and costs.

**Regret**	**E**	**IV**	**UV**	**AV**	**TEC**	**OEC**	**LoVA**	**EC**
Commission/omission	0.062	0.031	−0.016	0.047	−0.011	−0.056	−0.032	−0.032
Regret intensity	−0.265^*^	−0.126	−0.120	0.029	0.172	0.091	0.163	0.351^***^
Intrusive thoughts	−0.053	0.001	−0.095	−0.129	0.100	0.196	0.073	0.077
Total *R*^2^	0.101	0.018	0.037	0.017	0.063	0.078	0.051	0.168

E, expectancy for success; IV, intrinsic value; UV, utility value; AV, attainment value; TEC, task effort cost; OEC, outside effort cost; LoVA, loss of valued alternative; EC, emotional cost. Regret of commission (coded as 1) or omission (coded as 2).

* *p* < 0.05,

** *p* < 0.01;

*** *p* < 0.003 (Bonferroni corrected value), standardized betas are reported.

**Table 3:** Study 1: predicting achievement goals and emotions.

**Regret**	**MAP**	**MAV**	**PAP**	**PAV**	**PA**	**PD**	**NA**	**ND**
Commission/omission	−0.044	−0.029	−114	−0.045	0.009	−0.143	−0.027	0.030
Regret intensity	0.101	0.312^**^	0.357^***^	0.390^***^	−0.196	−0.230^*^	0.381^***^	0.306^**^
Intrusive thoughts	−0.193	−0.209	−0.140	−0.134	−0.037	−0.048	−0.067	−0.076
Total *R*^2^	0.023	0.059	0.101	0.108	0.050	0.078	0.120	0.068

MAP, mastery approach goals; MAV, mastery avoidance goals; PAP, performance approach goals; PAV, performance avoidance goals; PA, positive-activated emotions; PD, positive-deactivated emotions; NA, negative-activated emotions; ND, negative-deactivated emotions. Regret of commission (coded as 1) or omission (coded as 2).

* *p* < 0.05;

** *p* < 0.01;

*** *p* < 0.003 (Bonferroni corrected value), standardized betas are reported.

For the regressions that explored achievement goals and emotions, results indicated that ratings of commission or omission, regret intensity, and intrusive thoughts associated with the regret explained a significant amount of performance approach goals [*R*^2^ = 0.101, *F*_(3, 133)_ = 4.995, *p* = 0.003], performance avoidance goals [*R*^2^ = 0.108, *F*_(3, 133)_ = 5.350, *p* = 0.002], and negative-activated emotions [*R*^2^ = 0.120, *F*_(3, 132)_ = 5.996, *p* < 0.001] but did not significantly predict mastery approach goals [*R*^2^ = 0.023, *F*_(3, 133)_ = 1.025, *p* = 0.384], mastery avoidance goals [*R*^2^ = 0.059, *F*_(3, 133)_ = 2.766, *p* = 0.044], positive-activated emotions [*R*^2^ = 0.050, *F*_(3, 131)_ = 2.287, *p* = 0.082], positive-deactivated emotions [*R*^2^ = 0.078, *F*_(3, 132)_ = 3.743, *p* = 0.013], and negative-activated emotions [*R*^2^ = 0.068, *F*_(3, 133)_ = 3.256, *p* = 0.024]. Similar to the expectancy, values, and costs analyses, only emotional intensity was a statistically significant predictor. Emotional intensity predicted of performance-approach goals (β = 0.357, *p* = 0.001), performance-avoidant goals (β = 0.390, *p* < 0.001), and negative-activated emotions (β = 0.381, *p* < 0.001).

## Discussion

This study provides initial evidence for (1) the types of salient academic regrets that college students experience, (2) how these regrets differ in terms of intensity, intrusive thoughts, and ratings of omission or commission, and (3) correlates of regrets.

Results contribute to research by advancing a finer grained exploration of the diversity of regrets found specifically in academic contexts and therefore provide a more nuanced understanding of the most common answer to the question “what is your most severe life regret?” (Roese and Summerville, 2005).

Although seven types of academic regrets were identified, the intensity and intrusive thoughts associated with the regrets do not seem to vary based on the type of regret reported. Regrets did however differ in ratings of omission or commission. Regrets categorized as a missed opportunity were more likely than other regrets to be considered a regret of omission.

In contrast to prior research on life regrets, no differences in intensity were found between regrets of commission or omission, but regrets of commission were associated with a different maladaptive outcome, namely slightly higher levels of intrusive thoughts. While inconsistent with Gilovich and Medvec's (1994) original study, these results align more closely with the results of a recent replication study conducted by Richardson and Gilovich (2023), which found that participants were more troubled by regrets of commission than omission in the short term and reported no differences between commissions and omissions in the long term. An alternative explanation for these results may be due to the focus of the present study on academic regrets rather than life regrets.

Whether a regret was categorized as a commission or an omission, and the amount of intrusive thoughts associated with a regret were unrelated to achievement goals, expectancy, values, costs, and emotions, however regret intensity consistently predicted these outcomes in the expected direction. Generally, the lower the regret intensity, the more adaptive the outcome. These results provide initial support for the relevance of students' prior academic regrets on current motivational and emotional experiences and offer additional support for Buchanan et al.'s (2016) assertion that it is important to distinguish between the “maladaptive” affective and “functional” cognitive components of regrets. Although the cognitive component measured (i.e., intrusive thoughts) did not relate to adaptive outcomes, it also did not relate to maladaptive outcomes, and the affective component (i.e., regret intensity) tended to predict less desirable outcomes. Overall, these results can be explained within CVT (i.e., regrets are retrospective outcome emotions related to subsequent emotions and appraisals, Pekrun, 2006) and SEVT (i.e., regrets are an affective memory that relates to expectancy and values, Eccles and Wigfield, 2020).

Results from Study 1 provide insight into the types of regrets experienced and suggest that academic regrets relate to general academic experiences (e.g., regret intensity predicts general expectancy for success). Study 2 extends these results by focusing on academic regrets and relations to motivation and emotions situated in a specific undergraduate course. It remains unclear what types of regrets are reported among undergraduate students enrolled in a CS course, and if academic regrets relate to experiences situated in a particular course (e.g., does regret intensity predict expectancy for success in an undergraduate CS course?).

## Study 2

In Study 2, we replicate and extend previous results by exploring the same research questions, with a similar research design, measures, and analyses, but in a more specific context of a large undergraduate CS course.

### Participants and procedures

Participants were 128 undergraduate students recruited from the same university as those in Study 1. The sample consisted of 71 men, 53 women, one transgender student, and three students who did not report their gender. The mean age of participants was 19.93 (*SD* = 3.47). Racial or ethnic identity consisted of 59 Asian or Asian American, 24 Hispanic or Latinx, 16 White and non-Hispanic, 12 Black or African American, 10 as mixed-race, and 7 as “other.” Approximately 57% of participants reported majoring in CS, 23% undeclared, 6% a social science, 4% mathematics, 3% English, 8% other, and 2% did not report a major.

Participants were recruited from a large introductory to CS course, which is a pre-requisite for a major in CS, and an elective for STEM majors. Surveys were administered online at a single timepoint to all participants who provided consent. Questionnaires were organized in the same order as Study 2, and participants were compensated with a $10 online gift code. Following results from QCA and to maintain consistency with Study 1, four participants who reported either “no-regret” or a “non-academic regret,” and six students who reported regrets categorized as “competing commitments” were removed from the sample, leading to a total sample size of 118.

### Measures

Except for a different measure of achievement goals, all measures in Study 2 were identical to the measures used in Study 1. However, in contrast to Study 1, participants were instructed to think about their experiences in the in-class lecture portion of their current CS course while answering the achievement goals, expectancy, value, costs, and emotions questionnaires. Furthermore, items were slightly modified to reflect the specific course context (e.g., the sample expectancy item from Study 1: “I'm certain I can master the skills taught in my classes,” was modified to “I'm certain I can master the skills taught in this class”). The academic regrets measure was not modified and focused on participants' most severe academic regret.

#### Achievement goals

Mastery-approach, mastery-avoidance, performance-approach, and performance-avoidance goals were measured using the Achievement Goal Questionnaire-Revised (AGQ-R; Elliot and Murayama, 2008). Each goal was assessed with three items. A sample item for mastery-approach goals is “My aim is to completely master the material presented in this class” (α = 0.79), for mastery-avoidance goals is “My aim is to avoid learning less than I possibly could” (α = 0.63), for performance-approach goals is “My aim is to perform well relative to other students” (α = 0.86), and a sample item for performance-avoidance goals is “My aim is to avoid doing worse than other students” (α = 0.82).

## Results

### Preliminary analyses

Descriptive statistics, reliability, and correlations can be found in Table 1. Skewness ranged from −0.67 to 1.06 and kurtosis ranged from −0.84 to 1.16, both within acceptable ranges for normality (e.g., Byrne, 2010; Hair et al., 2010). The pattern of correlations within scales is largely consistent with those reported in Study 1. Despite many similarities, there are some evident differences in relations identified in the two studies. In particular, the relations between achievement goals and expectancy, values, and costs, and between achievement goals and emotions were inconsistent, possibly due to the different measures of achievement goals (i.e., PALS vs. AGQ-R). Especially relevant for the present study was the lack of relations between regret intensity or intrusive thoughts, and achievement goals, expectancy, value, costs, and emotions. Whereas, in Study 1 regret intensity and intrusive thoughts consistently related to these outcomes (e.g., 13 out of 16 possible correlations between regret intensity and outcomes were statistically significant), no statistically significant correlations were identified in Study 2. Therefore, in Study 2, regret intensity and intrusive thoughts did not correlate significantly with achievement goals, expectancy, value, costs, or emotions.

### RQ 1: Qualitative content analysis of open responses

The results of QCA suggested that the academic regrets reported by participants could be reliably coded into the same eight themes that emerged in the previous study (Cohen's Kappa = 0.861). Although the same themes were identified, the frequency of the themes differed slightly (see Supplementary Table 1S). The most reported academic regret was low effort, persistence, or performance (36.6%), followed by taking the wrong class, program, or school (21.1%), poor timing (14.8%), a missed opportunity (7%), regrets associated with a discrete event or decision (7%), not seeking help (5.5%), competing commitments (4.7%), and no regret/non-academic regret (3.1%).

### RQ 2: Differences in intensity, intrusive thoughts, and ratings of omission/commission

Regret intensity and intrusive thoughts did not differ based on the regret theme [Wilks' Λ_(110, 2)_ = 0.938, *p* = 0.706, η^2^ = 0.032], and intrusive thoughts did not differ between regrets categorized as omissions or commissions [*F*_(1, 113)_ = 1.769, *p* = 0.186, η^2^ = 0.015]. However, regret intensity did differ between regrets of omission and commission [*F*_(1, 114)_ = 5.297, *p* = 0.022, η^2^ = 0.045], suggesting that participants who shared regrets of omission (*M* = 2.39, *SD* = 1.05) reported slightly lower levels of regret intensity than participants who identified their regrets as commission (*M* = 2.82, *SD* = 0.93). A chi-square test revealed no differences in ratings of omission or commission across the six regret themes [i.e., missed opportunity; specific event or behavior; wrong class, program, or school; effort, persistence, or performance; timing; help seeking; $\chi_{\left(5\right)}^{2}$ = 6.787, *p* < 0.237]. Therefore, participants were just as likely to categorize their regret as an omission or commission regardless of the regret theme.

### RQ 3: Predicting achievement goals, emotions, expectancy, value, and costs with regret intensity, intrusive thoughts, and ratings of omission/commission

The same series of multiple regressions used in Study 1 were conducted to explore how regret intensity, intrusive thoughts, and ratings of regrets as an omission or commission relate to achievement goals, expectancy, values, costs, and emotions (see Tables 4, 5; see Supplementary Tables 4S, 5S for results when controlling for age and gender). None of the regressions were statistically significant, indicating that these regret characteristics do not predict course specific motivational and emotional outcomes.

**Table 4:** Study 2: predicting expectancy, values, and costs.

**Regret**	**E**	**IV**	**UV**	**AV**	**TEC**	**OEC**	**LoVA**	**EC**
Commission/omission	−0.045	−0.065	−0.130	−0.174	−0.111	−0.099	−0.095	−0.122
Regret intensity	−0.056	0.044	−0.160	−0.121	−0.074	0.118	0.108	0.020
Intrusive thoughts	−0.049	0.086	0.042	−0.039	0.151	−0.029	−0.055	0.104
Total *R*^2^	0.009	0.021	0.029	0.042	0.027	−0.002	0.019	0.006

E, expectancy for success; IV, intrinsic value; UV, utility value; AV, attainment value; TEC, task effort cost; OEC, outside effort cost; LoVA, loss of valued alternative; EC, emotional cost. Regret of commission (coded as 1) or omission (coded as 2). ^*^p < 0.05; ^**^p < 0.01; ^***^p < 0.003 (Bonferroni corrected value), standardized betas are reported.

**Table 5:** Study 2: predicting achievement goals and emotions.

**Regret**	**MAP**	**MAV**	**PAP**	**PAV**	**PA**	**PD**	**NA**	**ND**
Commission/omission	−0.113	0.076	−0.105	−0.088	−0.052	0.139	−0.087	0.006
Regret intensity	−0.108	0.080	0.201	0.199	0.105	−0.136	0.040	−0.002
Intrusive thoughts	0.042	−0.192	−0.245^*^	−0.108	0.031	−0.016	0.097	0.029
Total *R*^2^	0.017	0.005	0.051	0.036	0.016	0.049	0.027	0.001

MAP, mastery approach goals; MAV, mastery avoidance goals; PAP, performance approach goals; PAV, performance avoidance goals; PA, positive-activated emotions; PD, positive-deactivated emotions; NA, negative-activated emotions; ND, negative-deactivated emotions. Regret of commission (coded as 1) or omission (coded as 2). ^*^p < 0.05; ^**^p < 0.01; ^***^p < 0.003 (Bonferroni corrected value), standardized betas are reported.

## Discussion

This study extends the results from Study 1 by investigating the same set of research questions in the more specific context of a large undergraduate CS course. Results indicated that the same coding manual could be used to reliably categorize students' salient academic regrets. The three most frequently reported regrets were (1) low effort, persistence, or performance, (2) taking the wrong class, program, or school, and (3) poor timing. Together these three categories of regrets accounted for 72.5% of the regrets reported in this sample and highlight the common types of affective memories experienced by these students.

Similar to the results reported in Study 1, regret themes were unrelated to levels of regret intensity, or the amounts of intrusive thoughts experienced, and intrusive thoughts did not differ between regrets of omission and commission. In contrast to the results reported in Study 1, no differences were identified for regrets of omission or commission across the regret themes (e.g., missed opportunities and poor timing were equally likely to be considered regrets of omission or commission). Furthermore, regrets of omission or commission did not differ in levels of intrusive thoughts but regrets of omission were associated with slightly lower levels of regret intensity than regrets of commission. This result provides additional support for Richardson and Gilovich (2023) by suggesting that regrets of omission may not be as troubling as previously suggested. However, it is critical to interpret these findings with caution given the focus on specific academic regrets and not more general life regrets.

Overall, regrets of omission or commission, regret intensity, and intrusive thoughts were unrelated to expectancy, values, costs, achievement goals, and emotions. The most striking differences between these results and those reported in Study 1 are the lack of relations identified between regret intensity and these outcomes. In Study 1, regret intensity predicted 8 of the 16 outcomes at a *p*-value of at least 0.05 (4 of 16 at a *p*-value of 0.003), whereas in Study 2 regret intensity predicted only 1 of 16 outcomes at a *p*-value of 0.05. This illuminates the critical importance of considering the situated nature of motivational and emotional constructs and highlights the relevance of revising expectancy-value theory (Wigfield and Eccles, 2000) into *situated* expectancy-value theory (Eccles and Wigfield, 2020).

## General discussion

The first set of results highlight the variety of academic regrets that students report, as well as a consistent frequency of different types of regrets between the two studies. Across both studies, students' academic regrets were reliably coded into seven distinct themes with the three most common types of regrets being (1) taking the wrong class, program, or school, (2) low effort, persistence, or performance, and (3) poor timing. In Study 1, taking the wrong class, program, or school was the most frequent regret (25.3%) whereas in Study 2, low effort, persistence, or performance was the most reported regret (36.6%). One possible explanation for these differences could be that students who are beginning a degree in CS are more satisfied with their disciplinary choices to date due to consistent encouragement to pursue a degree in CS. In contrast, the majority of participants in Study 1 were recruited from the School of Education and may be more regretful of their academic choices thus far. Students may have become accustomed to seeing headlines about the positive job outlooks and salaries for CS graduates (e.g., Espinel, 2018; Kennedy, 2015) or high burnout and low retention among teachers (e.g., Dill, 2022; Lane, 2022; Marken and Agrawal, 2022). These types of messages may shape students' thinking toward more upward (e.g., “I should have selected a less stressful career path”) or downward (e.g., “At least I'm not becoming a teacher”) counterfactuals (Gilovich and Medvec, 1995), which would explain the differences in the most common regret reported in the two studies. Within SEVT and CVT these messages could be considered antecedents of students' academic regrets and framed as part of the cultural milieu or cultural context (Eccles and Wigfield, 2020; Pekrun, 2024).

Another explanation for why achievement related concerns (e.g., effort, persistence, or performance) were reported more frequently in Study 2 than in Study 1 may relate to the perceived difficulty of completing a degree in CS. Prior research suggests that CS degrees are perceived as especially difficult (e.g., Leslie et al., 2015), which could make regrets associated with achievement related concerns more salient among participants who are enrolled in a CS program. Identifying the salient academic regrets reported by college students contribute to the regrets literature by providing a finer grained analysis of the types of academic regrets that students may report. Regret ranking studies tend to focus on coding general life regrets into broad categories such as education, career, romance, parenting, self, leisure, finance, family, and health, but then do not explore possible sub-themes contained within these categories (Roese and Summerville, 2005; Wrosch et al., 2005). By deepening our understanding of the types of regrets that students experience, we can begin to conceptualize ways to help them avoid experiencing these regrets, or to make sure that these regrets are dealt with in a positive manner.

The second set of results provide a deeper understanding of characteristics of academic regrets, including intensity, intrusive thoughts, and ratings of regrets as omissions or commissions. To date, research has focused on characteristics of general life regrets, but has not yet investigated the characteristics of academic regrets, therefore this set of results was largely exploratory. Results across both studies suggested that the types of regrets that students experience are unrelated to regret intensity or amounts of intrusive thoughts. Therefore, students who reported a regret associated with poor timing reported similar levels of regret intensity as students who reported a regret associated with not seeking help. This lack of differences may be due to the measurement approach used in the present study. Asking participants to report their most severe academic regret may have reduced the amount of variability in regret intensity and intrusive thoughts among the different types of regrets shared. Although regret intensity and intrusive thoughts did not differ across regret themes, they did differ between regrets of omission and regrets of commission. In Study 1, regrets of commission were associated with more intrusive thoughts and in Study 2, regrets of commission were associated with higher regret intensity. These results suggest that students report more maladaptive experiences associated with an action taken as opposed to something not done and contribute to the mixed results in this area. Gilovich and Medvec's (1994) original work suggested that regrets of omission were more troubling than regrets of commission, however in a follow up study, Richardson and Gilovich's (2023) did not replicate this finding. While participants reported that regrets of commission were perceived as more troubling in their replication than regrets of omission, this was only the case for short term regrets, with no differences being reported for more long-term regrets. Similarly, there were no differences between regrets of commission and omission for regret intensity in Study 1 or for intrusive thoughts in Study 2. Untangling this pattern of results is difficult and will require targeted research on potential moderators such as regret timeframes (i.e., short-term and long-term), regret category (e.g., general life or academic regrets), outcomes (e.g., troublesomeness, intensity, intrusive thoughts), as well as careful distinctions between affective and cognitive components of regrets (Buchanan et al., 2016).

The third set of results begin to connect academic regrets with motivational and emotional outcomes. Across both studies, regrets of commission or omission, and intrusive thoughts associated with regrets were unrelated to motivational and achievement outcomes. However, regret intensity predicted various outcomes in Study 1. Specifically, higher regret intensity was associated with higher levels of emotional costs, performance approach goals, performance avoidance goals, and negative activated emotions. This suggests that regret intensity is an antecedent of generally maladaptive outcomes, which begins to bridge research on regrets and theories of motivation and emotion (i.e., Pekrun, 2006; Wigfield and Eccles, 2000). Interestingly, these results were not replicated in Study 2, where domain specific motivational and emotional outcomes, situated in an undergraduate CS class were assessed. One explanation for the distinct results may be due to differences in levels of generalizations between the two studies, with general academic regrets aligning more closely to general academic outcomes (Study 1) than course specific outcomes (Study 2). Similar explanations have been proposed in prior research on hierarchical models of self-concept and enjoyment (Goetz et al., 2006; Marsh and Yeung, 1998; Shavelson et al., 1976). Therefore, since academic regrets and the motivational and emotional outcomes in Study 1 were both located at the same “academic” level they should be more strongly related than relations between academic regrets located on the “academic” level and outcomes located at the “class” level. Taken together, these results highlight the relevance of considering the situative nature of motivational and emotional constructs (Eccles and Wigfield, 2020; Pekrun and Marsh, 2022).

### Implications for theory

The results from these studies help situate academic regrets within current research and theory on motivation and emotion in educational psychology. Prior to discussing connections to theories that were hypothesized to be relevant a priori (i.e., CVT and SEVT), we first discuss connections to emergent themes that arose from the qualitative content analyses. The salient and most severe academic regrets that students shared provide an initial bridge between regrets that students may experience in educational contexts and current research foci in the field such as identity formation, academic engagement, or self-regulated learning. One of the most frequently reported academic regrets involved taking the wrong class, program, or attending the wrong school, which connects with attainment value within SEVT, and Marcia's (1993) impactful work on identity development. While attainment value was originally framed as “the needs and personal values that an activity/behavior or task fulfills” (Eccles, 2009, p. 83), Eccles' has also conceptualized attainment value as engagement in an individual or collective identity-affirming task. Consequently, taking the wrong program may reflect a regret associated with misalignment between one's personal identity and current roles. Similarly, according to Marcia (1993), identity is shaped through commitment, which consists of identifying personal goals and values, and exploration, which involves searching for and analyzing information that can help make meaningful life decisions. Therefore, regrets such as selecting the wrong program may emerge among foreclosed students who psychologically committed to an area of study without adequate exploration or students in moratorium, who are currently exploring programs but have not yet committed.

Another frequently reported academic regret consisted of low effort, poor persistence, or performance concerns, which relates to one of the most popular areas of research in educational psychology, namely engagement (Fredricks et al., 2004). Engagement consists of behavioral, cognitive, affective, and agentic dimensions that predict critical and long-term outcomes such as motivation, academic achievement, and retention (e.g., Reeve and Tseng, 2011; Reschly and Christenson, 2012; Tao et al., 2022). Regrets associated with prior effort, persistence, and achievement may emerge from a student's recognition of past engagement related failures (e.g., not putting in enough effort in high school), or the realization that lack of engagement in the past has limited opportunities in the present (e.g., I should have learned more in my introductory courses).

A final consistently reported regret that intersects with prior research involves poor timing. Poor timing was characterized by a broad set of timing related failures, such as not completing a degree when younger, taking too long to complete a degree, or taking too much time off between degrees. These regrettable experiences may relate to students' past struggles to self-regulate their learning. Self-regulated learning refers to how “learners systematically activate and sustain their cognitions, motivations, behaviors, and affects” (Schunk and Greene, 2017, p. 1). From planning, monitoring, and controlling time use found in many theories of self-regulated learning (e.g., Efklides, 2011; Winne and Hadwin, 1998), to empirical research on time management in education (e.g., Ranellucci et al., 2015), managing time is a fundamental part of self-regulated learning. As such, regrets associated with poor timing may emerge due to suboptimal goal planning, monitoring time use, or controlling competing time commitments.

Turning now to the theoretical frameworks that guided this project, results from the two studies provide initial support for the role of academic regrets in both CVT (Pekrun, 2006) and SEVT (Eccles and Wigfield, 2020). In Study 1, regret intensity predicted emotional costs, performance approach and performance avoidance goals, and negative activated emotions, whereas in Study 2, regret intensity did not predict any of the measured outcomes. What makes this especially interesting is the contextual and temporal distance between the academic regrets that students may report and the outcomes that are predicted. Students may be reporting a regret associated with not putting in enough effort years earlier in high school, which then relates to their performance goals and emotional experiences years later in college. At first, it might sound surprising that such an effect would be detectable, but from a theoretical perspective, if academic regrets are considered to be a type of affective experience associated with significant life events which are stored in long-term memory and surface based on environmental cues (see Pillemer, 2001), then students' most severe academic regrets should relate to current experiences. When students' most severe academic regrets are recalled due to environmental cues (e.g., when facing a new decision about what class to take, struggling with achievement, or taking longer to complete an assignment), they may impact important processes that also arise in these environments (e.g., achievement goals).

The purpose of Study 2 was to provide a conceptual replication with a goal of identifying the generalizability and boundary conditions of the results (see Plucker and Makel, 2021). The rationale for including the second study follows from recent calls in the literature to consider the situative nature of motivational and emotional constructs (Eccles and Wigfield, 2020; Pekrun and Marsh, 2022). According to Pekrun and Marsh's (2022) 2 × 2 model of situational variation and study designs, this project consisted of cross-sectional comparative studies that investigated variation in context. Meanwhile, according to the latest revision of SEVT, unique lived experiences shape approaches to behavioral options at specific time points and locations (Eccles and Wigfield, 2020). Tracing the path from the left side of SEVT to the right side, affective memories associated with significant academic regrets from ones' past may guide future beliefs, decisions, and behaviors situated in some settings (e.g., general academic contexts), but not others (e.g., a specific CS course). The results from these two studies highlight the critical importance of considering the situative nature of not only motivational and emotional processes, but also of the relations between affective memories and motivational and emotional processes. These findings point to a new, fruitful, and theoretically aligned avenue to explain students' academic experiences and also provide relevant implications for practice.

### Implications for practice

When discussing implications for practice it is important to contextualize the functional basis of regrets. Although colloquially, regrets are often considered maladaptive, they play an important role in guiding regulation and future improvements (see Epstude and Roese, 2008). We should be hesitant to recommend strategies to avoid regrets altogether but rather focus on informed decisions about possible future regrets and healthy regulation of past regrets.

By anticipating regrets, students can make informed decisions and avoid long-lasting high-intensity regrets. There is strong evidence that anticipated regrets predict both behavioral intentions and actual behaviors (Sandberg and Conner, 2008). As such, helping students reflect on potential regrets can help guide important academic choices or behaviors. Students can get support for big academic decisions by consulting with peers, instructors, or academic advisors, and becoming familiar with common academic regrets that students have reported in similar situations. Results from these two studies suggest that students may want to focus on (1) taking the *right* class, program, or school, (2) investing in *high* effort, persistence, or performance, and (3) *appropriate* timing of choices. Institutions can support their students by making career counselors available to incoming students to help with program and course selection and offering a “learning to learn” course to help students' maintain engagement and manage their time (e.g., Wolters et al., 2023). Furthermore, there is some evidence that framing interventions, such as framing grade allocation as a loss, can impact anticipated regrets (Tifferet, 2020). However, because completely avoiding future regrets may be impossible, it is also important to help students regulate past academic regrets.

There are various emotion regulation strategies that can be useful for down-regulating negative emotions (Gross and Thompson, 2007). Guided by Gross's (1998) process model of emotion regulation, regrets may be regulated through situation selection, situation modification, attentional deployment, cognitive change, and response modulation. Broadly, reappraisal, which entails cognitively reframing a negative situation as something more positive, is more adaptive than suppression, which involves concealing expressions of emotions (Gross, 2001). Therefore, students should be encouraged to reappraise their academic regrets as learning opportunities that can be used to guide future choices and behaviors.

### Limitations and future directions

There are a few relevant limitations that should be considered. In particular, a much deeper exploration of students' academic regrets is needed. In the present studies, inductive QCA was used to identify salient academic regrets reported by students. This approach was adapted from the life regrets literature and involved reporting regrets in an open-ended question and then completing Likert scale items about these regrets (e.g., Wrosch et al., 2005, 2007; Wrosch and Heckhausen, 2002). This approach is constrained because it assumes that students only have a single pertinent regret and that regrets are stable across time and situation. The nature, content, and salience of regrets depends on our current situation, including motivational and emotional states, which highlights the importance of exploring reciprocal effects (e.g., how current academic emotions predict the types of regrets reported). Future research can address these limitations by including the option to report multiple regrets (e.g., “what are your three most severe academic regrets?”), by assessing academic regrets at different timepoints during the semester (e.g., at the beginning of the semester, during exam periods, and during summer break), by having students provide a deeper explanation of their academic regrets, or by asking students to report their regrets after inducing a particular emotion. Another limitation of the results is that it remains unclear why, despite reporting similar themes of regrets between the two studies, regret intensity predicted motivational and emotional outcomes in Study 1, but not in Study 2. Future research can investigate the relations between students' academic regrets and outcomes across various disciplines and further extend these results by exploring a broader set of outcomes. In particular, based on the emergent themes identified, it may be beneficial for future studies to explore the relations between academic regrets and students' identity formation, academic engagement, and self-regulated learning.

The results from this study raise important theoretical and practical questions. Within SEVT (Eccles and Wigfield, 2020), how do students' cultural milieu (e.g., stereotypes), personal characteristics (e.g., ethnicity), and previous achievement-relate experiences (e.g., failure) shape the types and severity of academic regrets reported? Within CVT (Pekrun, 2006), how does academic regret activation levels relate to students' learning and achievement outcomes? Meanwhile, the effectiveness of emotion regulation strategies and interventions targeting academic regrets need to be evaluated. For instance, given the unusually cognitively laden nature of academic regrets (Gilovich and Medvec, 1995), is cognitive-reappraisal sufficient for reducing potential negative (or maximizing positive) experiences and outcomes of academic regrets?

## Data Availability

The datasets presented in this article are not readily available because IRB permission was not granted. Requests to access the datasets should be directed to john.ranellucci@uottawa.ca.

## Footnotes

^1^One item was removed due to poor factor loading.

^2^Note. It is important to interpret the adequate to poor fit based on the context of this study. McNeish and Wolf (2023) advise that “the meaning of fit indices varies based on a complex interaction of model characteristics like factor reliability, number of items, and number of factors” (p. 61), as well as sample size. While all measure reported are reliable, the measurement models were complex (e.g., degrees of freedom ranged from 53 to 183) in relation to the sample sizes (*N*_*study*1_ = 138; *N*_*study*2_ = 118).

^3^To provide a more conservative approach due to multiple comparisons and the exploratory nature of this study, a Bonferroni correction was conducted, where the *p*-value of 0.05 was divided by the number of regressions (16) resulting in a *p*-value cut-off of 0.003.
