# Supplementary material for: Investigating the content and correlates of undergraduate students' academic regrets
Source: Front Psychol. 2025 Apr 17;16:1436323. doi: 10.3389/fpsyg.2025.1436323 (PMC12043591; doi:10.3389/fpsyg.2025.1436323)
Supplement: Supplementary file 1 [file Data_Sheet_1.pdf]

Supplemental materials

Table 1S. Academic regret coding manual with frequencies

Code	Description and Example	Study 1 N	Study 1 %	Study 2 N	Study 2 %
Wrong class, program, or school	Selecting the wrong classes, program, major, school, or career path. (e.g., I regret that I didn't study different subjects than the ones I chose in the past" or "My most severe regret was not pursuing medical school" or "I regret the most not knowing that I wanted to become a teacher earlier in my life" or "I wish I took more science and math classes"	39	25.3	27	21.1
Low effort, persistence, or performance	Not working hard enough, not getting better grades, not doing the required work, procrastinating, or giving up. (e.g., "I regret not doing better in my first few semesters of college" or "Not doing enough in undergrad to get a better grade" or "My most severe academic regret is sometimes not putting all my effort into my studying"	35	22.7	47	36.6
Poor timing	The timeframe, length, or decisions related to the timing of one's degree or studies. Related to taking time off or not taking time off school, not coming back to school earlier, not completing the degree when younger, or finishing degree too slowly. (e.g., "I regret taking time off from school when I was younger" or "My most severe regret is having taken a semester off from school" or "I regret doing my undergraduate degree in four years instead of taking a year or two off"	22	14.3	19	14.8
Missed opportunity	Missing a learning, social, or professional opportunity in school. (e.g., "I regret being way too concerned about assignments that I never got any real hands on experience" or "I regret not being active in school groups and activities in my undergrad")	17	11.0	9	7.0
Not seeking help	Seeking help from teachers, peers, or academic counsellors (e.g., "Not getting help for a class" or "I would have participated more in class and ask questions when I needed the most help" or "I regret not being well informed. I had trouble determining what courses I had to take for my major. I often got lost and confused when choosing classes. I wish I had more guidance. I wish I would have reached out for help sooner"	15	9.7	7	5.5
Discrete event or decision	A discrete event or behavior that occurred in a specific moment or during a short time frame. (e.g., "I was late for the exam due to over sleeping" or "In undergrad I was caught plagiarizing" or "Not filling out a credit/no credit form"	10	6.5	9	7.0
No/non-academic regret*	Reported "no regret" or a non-academic regret (e.g., "having a child too early")	9	5.8	4	3.1
Competing commitments*	Too many competing commitments or activities. (e.g., "My most severe regret as a student was packing too many things outside of school into my first semester's schedule," "My most severe regret was allowing my personal life to influence my academic life" or "My most severe regret in terms of school is having taken too many courses at once because as a result, I wasn't able to focus on each course as much as I needed to and ended up underperforming in each class"	5	3.2	6	4.7
Total	-	152	100	128	100

Note. * Not included in quantitative analyses due to not relevant for the current study or represents too few participants for analyses. Responses were independently coded by two coders based on the coding manual. Disagreements were resolved through discussion between the two coders.

Table 2S. Study 1: Predicting expectancy, values, and costs, controlling for gender, age, and university level

**Regret**	E	IV	UV	AV	TEC	OEC	LoVA	EC
Gender	-.080	-.012	.060	-.054	-.068	-.102	-.043	-.044
Age	.074	.076	.042	.098	-.177	-.101	-.136	-.292
UG/G	.172	.010	.146	.100	-.036	.078	.046	-.092
R^2	.086	.011	.037	.034	.047	.017	.020	.149
Commission/omission	.038	. 005	-.055	.014	-.014	-.064	.046	-.028
Regret intensity	-.235*	-.126	-.070	.071	.140	.082	.175	.261**
Intrusive thoughts	-.093	-.126	-.143	-.183	.116	.200	.102	.141
Total R^2	.138	.034	.073	.057	.100	.093	.083	.283

Note. E = expectancy for success, IV = intrinsic value, UV = utility value, AV = attainment value, TEC = task effort cost, OEC = outside effort cost, LoVA = loss of valued alternative, EC = emotional cost. Regret of commission (coded as 1) or omission (coded as 2). ***$p < .003$ (Bonferroni corrected value), **$p < .01$, *$p < .05$, standardized betas are reported

Table 3S. Study 1: Predicting achievement goals and emotions, controlling for gender, age, and university level

**Regret**	MAP	MAV	PAP	PAV	PA	PD	NA	ND
Gender	-.001	-.041	-.041	-.060	.157	.004	.016	.124
Age	.098	-.052	-.172	-.210*	.077	.228*	-.088	-.117
UG/G	.112	.026	-.113	-.086	.091	-.134	.068	.033
R^2	.025	.008	.086	.094	.046	.047	.012	.040
Commission/omission	-.071	-.036	-111	-.043	-.025	-.134	-.029	.016
Regret intensity	.156	.301**	.304**	.319**	-.146	-.189	.342***	.268*
Intrusive thoughts	-.243	-.215	-.097	-.086	-.090	-.056	-.075	-.068
Total R^2	.062	.062	.164	.128	.089	.102	.099	.090

Note. MAP = mastery approach goals, MAV = mastery avoidance goals, PAP = performance approach goals, PAV = performance avoidance goals, PA = positive-activated emotions, PD = positive-deactivated emotions, NA = negative-activated emotions, ND = negative-deactivated emotions. Regret of commission (coded as 1) or omission (coded as 2). ***$p < .003$ (Bonferroni corrected value), **$p < .01$, *$p < .05$, standardized betas are reported

Table 4S. Study 2: Predicting expectancy, values, and costs, controlling for gender, age, and university level

**Regret**	E	IV	UV	AV	TEC	OEC	LoVA	EC
Gender	.041	.012	-.022	.053	-.063	-.031	.030	-.012
Age	-.001	.085	-.004	.057	.051	.088	.016	-.089
R^2	.002	.009	.001	.009	.010	.012	.001	.007
Commission/omission	-.059	-.058	-.121	-.147	-.092	-.069	-.076	-.115
Regret intensity	-.017	.080	-.136	-.077	-.108	.108	.125	-.008
Intrusive thoughts	-.055	.070	.022	-.075	.147	-.045	-.074	.109
Total R^2	.009	.032	.023	.042	.030	.026	.018	.031

Note. E = expectancy for success, IV = intrinsic value, UV = utility value, AV = attainment value, TEC = task effort cost, OEC = outside effort cost, LoVA = loss of valued alternative, EC = emotional cost. Regret of commission (coded as 1) or omission (coded as 2). ***$p < .003$ (Bonferroni corrected value), **$p < .01$, *$p < .05$, standardized betas are reported

Table 5S. Study 2: Predicting achievement goals and emotions, controlling for gender, age, and university level

**Regret**	MAP	MAV	PAP	PAV	PA	PD	NA	ND
Gender	-.020	-.084	.068	-.054	.031	.148	.038	.138
Age	.005	-.068	-.122	-.018	.028	-.025	-.054	.028
R^2	.001	.008	.017	.007	.001	.037	.002	.017
Commission/omission	.123	.106	-.131	-.105	-.038	.094	-.081	-.002
Regret intensity	.080	.126	.155	.143	.154	-.142	.041	-.015
Intrusive thoughts	.090	-.244	-.212	-.077	-.024	.031	.094	.053
Total R^2	.013	.054	.063	.034	.023	.064	.026	.019

Note. MAP = mastery approach goals, MAV = mastery avoidance goals, PAP = performance approach goals, PAV = performance avoidance goals, PA = positive-activated emotions, PD = positive-deactivated emotions, NA = negative-activated emotions, ND = negative-deactivated emotions. Regret of commission (coded as 1) or omission (coded as 2). ***$p < .003$ (Bonferroni corrected value), **$p < .01$, *$p < .05$, standardized betas are reported

Table 6S. Fit indices for confirmatory factor analyses of measures

**Factor**	X^2	df	CFI	RMSEA	SRMR	Loadings
Study 1						
RI, IT	129.210	53	.937	.102	.049	> .457
E, IV, UV, AV	472.123	164	.860	.117	.083	> .616
TEC, OEC, LoVA, EC	280.526	129	.913	.092	.051	> .657
MAP, MAV, PAP, PAV	403.069	183	.863	.093	.073	> .433
PA, PD, NA, ND	175.300	84	.914	.089	.073	> .595
Study 2						
RI, IT	89.478	53	.960	.076	.040	> .467
E, IV, UV, AV	437.081	164	.844	.119	.069	> .603
TEC, OEC, LoVA, EC	301.738	129	.909	.107	.048	> .686
MAP, MAV, PAP, PAV	113.325	48	.894	.107	.069	> .419
PA, PD, NA, ND	143.758	84	.940	.078	.063	> .548

Note. RI = regret intensity, IT = intrusive thoughts, E = expectancy for success, IV = intrinsic value, UV = utility value, AV = attainment value, TEC = task effort cost, OEC = outside effort cost, LoVA = loss of valued alternative, EC = emotional cost, MAP = mastery approach goals, MAV = mastery avoidance goals, PAP = performance approach goals, PAV = performance

avoidance goals, PA = positive-activated emotions, PD = positive-deactivated emotions, NA = negative-activated emotions, ND = negative-deactivated emotions.
